# Case Report: A case of jejunal T-cell non-Hodgkin lymphoma with secondary bone involvement presenting as gastrointestinal perforation

**DOI:** 10.3389/fmed.2026.1748641

**Published:** 2026-03-25

**Authors:** Yuru Gao, Ting Sun, Jianrong Sun, Feng Lu, Tao Wang, Jiabin Han

**Affiliations:** 1 Intensive Care Unit, Binzhou Medical University Hospital, Binzhou, Shandong, China; 2 Department of Hematology, Binzhou Medical University Hospital, Binzhou, Shandong, China

**Keywords:** bone involvement, case report, enteropathy-associated T-cell lymphoma, gastrointestinal perforation, non-Hodgkin lymphoma

## Abstract

**Background:**

Enteropathy-associated T-cell lymphoma (EATL) is a rare and highly aggressive subtype of non-Hodgkin lymphoma (NHL) that is strongly associated with celiac disease. It frequently manifests with severe complications, including intestinal obstruction or perforation, which underscores its adverse prognosis. Due to its rarity, the pathogenesis of EATL remains unclear, and standardized treatment protocols are lacking, necessitating further research to optimize management.

**Case report:**

We present the case of a 69-year-old woman whose initial manifestation was gastroduodenal perforation and acute peritonitis. Two months later, she was readmitted due to a small intestinal perforation, requiring emergency partial small-bowel resection with side-to-side jejunojejunostomy. Postoperative histopathological examination confirmed the diagnosis of EATL. Furthermore, diagnostic work-up during her hospitalization revealed secondary bone involvement, a rare finding in this disease.

**Conclusion:**

This case illustrates that EATL can manifest as life-threatening gastrointestinal perforation, which often necessitates urgent surgical intervention. It underscores the diagnostic challenge posed by this malignancy, especially when presenting atypically with rare extranodal involvement such as bone metastasis. A high index of suspicion, repeated biopsies, and early multidisciplinary collaboration are crucial for timely diagnosis and management of this aggressive lymphoma.

## Introduction

The gastrointestinal (GI) tract represents a common extranodal site of involvement for non-Hodgkin lymphoma (NHL). However, primary GI-NHL is relatively uncommon and is associated with a poor prognosis. The most common pathological subtypes are diffuse large B-cell lymphoma (DLBCL) and marginal zone lymphoma, entities for which diagnosis, staging, and treatment strategies have advanced considerably over the past two decades. In contrast, enteropathy-associated T-cell lymphoma (EATL) is a rare and aggressive T-cell subtype whose management remains particularly challenging (1–3). Clinically, patients with EATL often present with abdominal pain, diarrhea, vomiting, and weight loss. Nearly half of the patients present with an acute abdomen (4), often mandating emergency exploratory surgery for lesion localization. A definitive diagnosis can only be established following histopathological examination. This report details the case of a 69-year-old woman with EATL that was complicated by bone involvement and who initially presented with GI perforation. The patient was admitted to our institution (Affiliated Hospital of Binzhou Medical University) in September 2025, with small intestinal perforation and acute diffuse peritonitis. Subsequent pathological examination confirmed the diagnosis of small intestinal NHL. Furthermore, during the diagnostic workup, secondary bone involvement by lymphoma was detected. The patient’s medical history, clinical presentation, physical examination findings, laboratory results, imaging studies, and histopathological analyses are presented in detail in this report.

## Case report

A 69-year-old woman was admitted to our institution in September 2025 with small intestinal perforation and acute diffuse peritonitis. The diagnosis of jejunal T-cell non-Hodgkin lymphoma with secondary bone involvement was established following a complex clinical course. The details of her medical history, clinical presentation, diagnostic workup, and therapeutic management are provided below.

### Clinical presentation and initial workup (May–August 2025)

The patient had a two-year history of rheumatoid arthritis, for which she was on a regimen of leflunomide, tofacitinib citrate, and loxoprofen sodium. For the preceding 6 months, she had reported persistent pain in the right lower limb and knee. Beginning in May 2025, she experienced recurrent episodes of paroxysmal abdominal pain.

On May 10, 2025, gastroscopy performed at a local hospital revealed severe chronic active inflammation with ulcer formation at the gastric angle. Histopathological analysis demonstrated a dense lymphocytic infiltrate in the lamina propria; however, immunohistochemistry (IHC) findings were inconclusive, and a repeat biopsy was therefore recommended.

**Figure 1:**
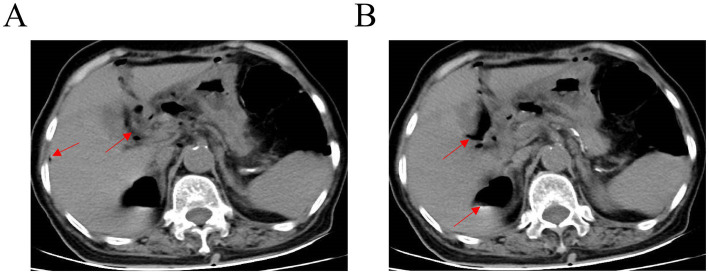
Abdominal CT images of the patient **(A,B)**. Abdominal CT scan showing free gas (red arrow) in the abdominal cavity.

**Figure 2:**
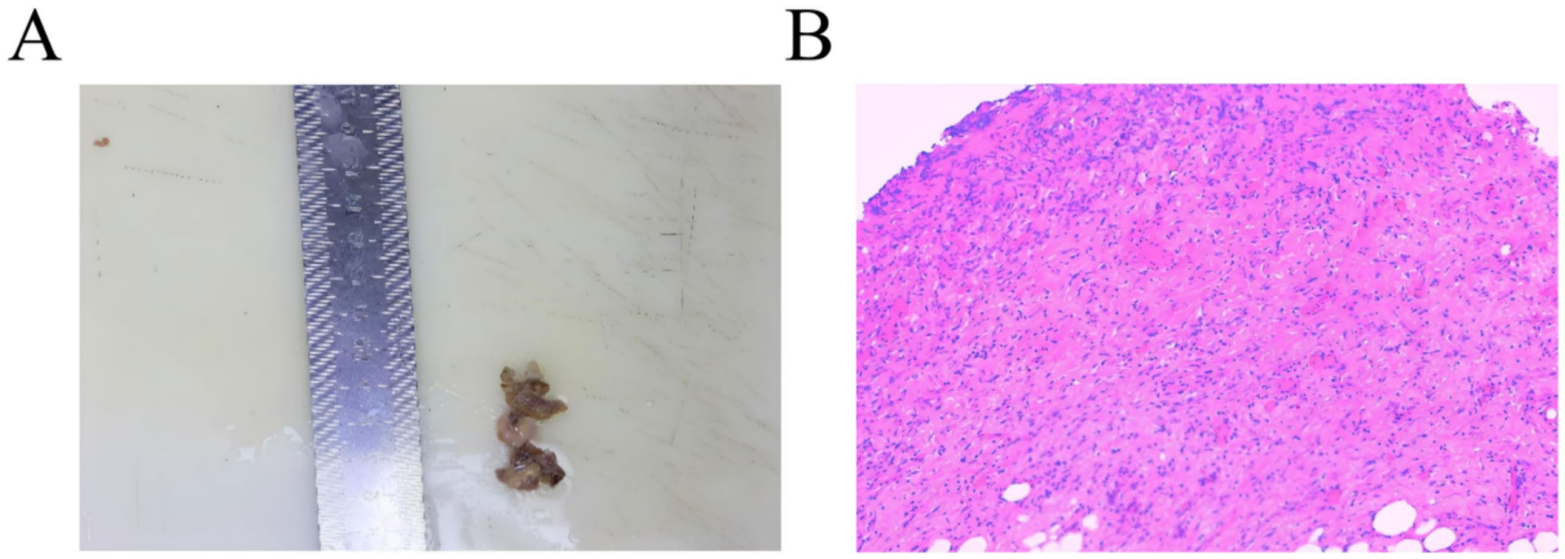
Histopathological examination (hematoxylin and eosin (H&E) staining, ×100): **(A)** Tissue surrounding the perforation site; **(B)** Submitted fibroadipose tissue showing vascular dilatation and congestion, dense infiltration by acute and chronic inflammatory cells, granulation tissue hyperplasia, and focal inflammatory necrosis.

On August 3, 2025, she presented to our institution due to worsening abdominal pain accompanied by nausea and vomiting. An emergency abdominal computed tomography (CT) scan demonstrated findings consistent with GI perforation (Figure 1). She subsequently underwent emergency laparoscopic repair of the gastric perforation and peritoneal lavage. Pathological examination of the perigastric tissue identified fibroadipose tissue with extensive acute and chronic inflammatory cell infiltration, granulation tissue hyperplasia, and focal necrosis (Figure 2).

### Hospital course and diagnostic challenges (September 2025)

**Figure 3:**
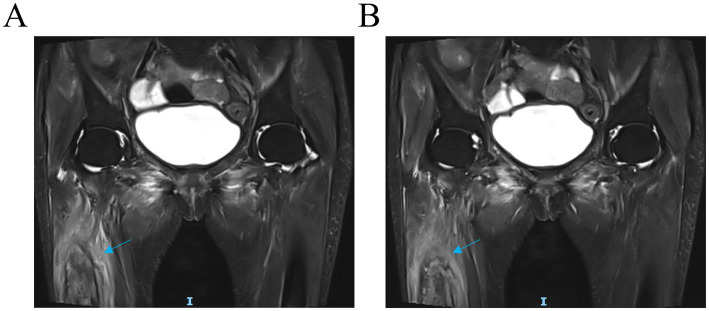
MRI of the patient’s knee and hip joints **(A,B)**. The noncontrast MRI scan demonstrated a patchy area of high signal intensity on fat-suppressed sequences within the femoral shaft, accompanied with localized cortical bone destruction and a possible periosteal reaction (blue arrow).

The patient was readmitted on September 12, 2025, owing to aggravated pain in the right limb and knee, which had severely limited her mobility. Magnetic resonance imaging (MRI) of the right hip and knee, obtained on September 13, revealed degenerative changes, joint effusion, and, notably, cortical bone destruction in the femoral shaft, suggestive of infection or neoplasm (Figure 3).

**Figure 4:**
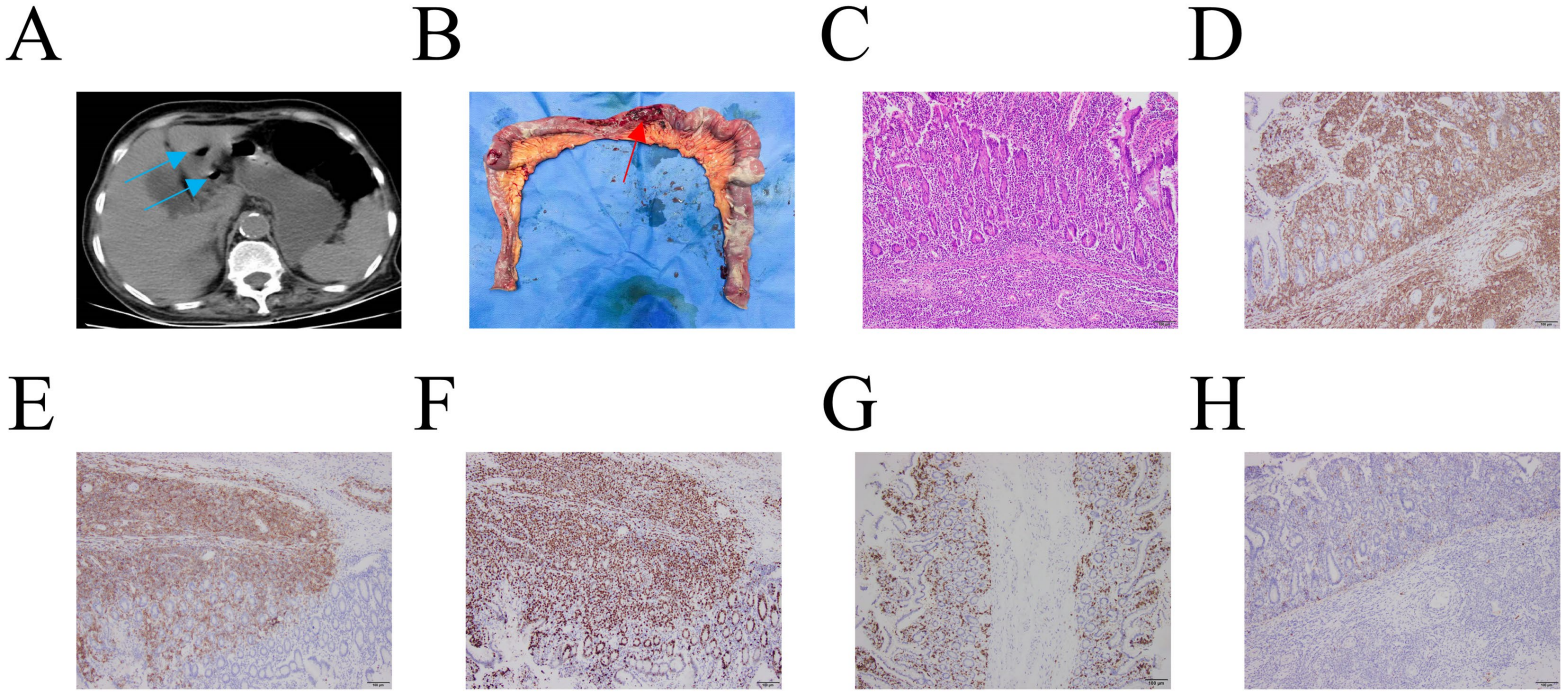
Abdominal CT, intraoperative findings, histopathological examination (H&E staining, ×100), and immunohistochemical analysis. **(A)** Abdominal CT scan showing free gas (blue arrow) in the abdominal cavity; **(B)** Intraoperative view revealing a small intestinal perforation (red arrow), with no evident mass identified; **(C)** Histopathological section demonstrating segmental transmural infiltration of tumor cells within the intestinal wall, associated with intestinal ulceration and perforation; immunohistochemical features of the tumor cells. Positive stains: CD3 **(D)**; CD30 **(E)**; Ki-67 **(F)**. Negative stain: CD5 **(G)**, CD56 **(H)**.

On September 15, 2025 (3 days later), she developed recurrent, severe abdominal pain accompanied by nausea and vomiting. A repeat abdominal CT scan confirmed the presence of a new GI perforation (Figure 4A).

### Surgical intervention and definitive diagnosis

On September 15, 2025, an emergency laparotomy was conducted. Intraoperative exploration revealed multiple jejunal perforations located approximately 50 cm distal to the ligament of Treitz, along with numerous scattered mass nodules throughout the small bowel (Figure 4B). Subsequently, a partial small-bowel resection followed by a side-to-side jejunojejunostomy was carried out.

Histopathological examination of the resected jejunal specimen revealed segmental, transmural infiltration by tumor cells, associated with ulceration and perforation (Figure 4C). Immunohistochemical (IHC) staining of the intestinal lesion showed positivity for CD3 (cytoplasmic), CD4 (partial), CD30, CD2, CD7, TIA-1, CD43, Granzyme B (partial), and Ki-67 (approximately 80%). The tumor cells were negative for CD56, CD8, CD20, CD79a, CD5, EMA, EBER, and CK. Collectively, these histopathological and immunohistochemical findings confirmed the diagnosis of enteropathy-associated T-cell lymphoma (EATL) (Figures 4D–H).

### Identification of secondary bone involvement

**Figure 5:**
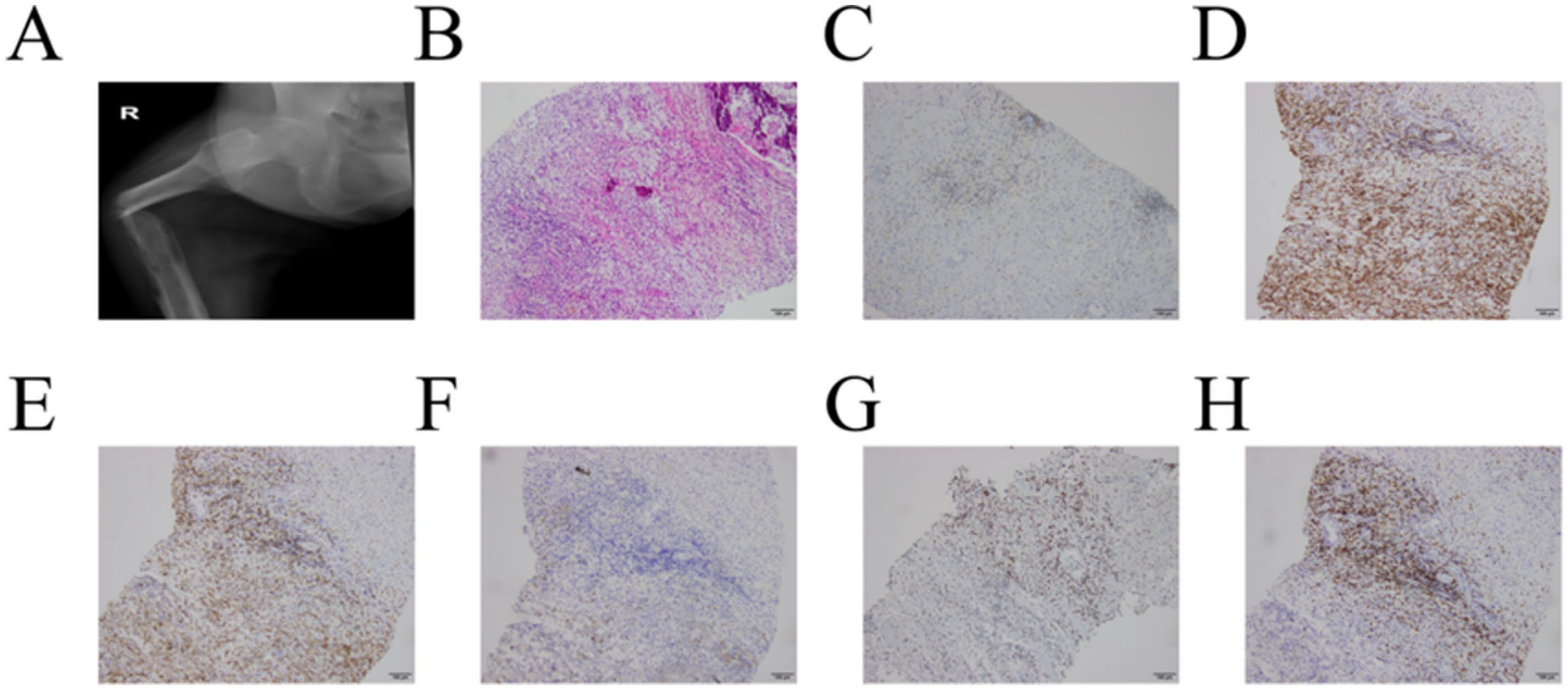
Radiograph of the right femur, histopathological examination (H&E staining, ×100), and immunohistochemical analysis. **(A)** Radiograph revealing a fracture in the middle-upper segment of the right femur. **(B)** Histopathological section showing the largely necrotic perifemoral tumor tissue, with only scant, scattered small malignant cells identified. Immunohistochemical features of the tumor cells. Positive stains: LCA **(C)**, CD2 **(D)**, CD3 **(E)**, CD30 **(F)**, Ki-67 **(G)**. Negative stain: CD5 **(H)**.

In the postoperative period, on September 17, 2025, the patient developed a spontaneous pathological fracture of the right femoral shaft. A bedside radiograph confirmed a fracture of the mid-to-upper femoral shaft (Figure 5A). Musculoskeletal ultrasonography, performed the following day (September 18), revealed cortical destruction accompanied by a surrounding hypoechoic mass. An ultrasound-guided biopsy of the perifemoral mass was subsequently undertaken. Histopathological analysis of the biopsy specimen demonstrated largely necrotic tumor tissue containing scattered malignant cells (Figure 5B). IHC profile of the bone lesion was as follows: CK (−), vimentin (partial +), LCA (+), CD3 (+), CD5 (−), CD20 (−), CD2 (+), CD30 (partial +), and Ki-67 (approximately 60%) (Figures 5C–H).

### Final diagnosis and management plan

Upon completion of the comprehensive diagnostic work-up, the case was reviewed at a multidisciplinary team (MDT) meeting. The patient’s prior non-contrast MRI had revealed localized bone destruction and a possible periosteal reaction. Furthermore, the femoral biopsy confirmed the involvement of EATL, confirming extranodal metastasis. Based on the confirmed osseous involvement, a diagnosis of stage IV disease was established, although subsequent bone marrow biopsy and positron emission tomography-computed tomography (PET-CT) were not conducted. According to the National Comprehensive Cancer Network (NCCN) guidelines, systemic therapy constitutes the primary management for stage IV lymphoma, with surgery indicated only for controlling local complications or in cases of oligometastatic disease. Moreover, considering the patient’s advanced age (69 years), poor performance status, and concomitant respiratory and circulatory failure, an extensive radical resection was considered to carry an unacceptably high risk of complications. Therefore, curative surgery was not considered a viable option. A palliative approach was therefore adopted, comprising brace fixation for the femoral fracture and general supportive care. The patient was discharged on September 30, 2025.

## Discussion

Acute abdominal pain is a common clinical presentation, with nearly one-third of affected patients requiring emergency intervention. Common etiologies of acute abdominal pain include GI obstruction, hemorrhage, ischemia, and visceral perforation (5). Additional causes encompass gynecological and urological disorders, abdominal trauma, and neoplastic diseases (6). GI perforation represents a life-threatening complication of GI lymphoma and most frequently involves the small intestine. Although chemotherapy is a common precipitating factor, perforation manifests prior to any treatment in approximately 44% of affected patients. Among the various subtypes of intestinal lymphoma, DLBCL is most frequently associated with intestinal perforation (7).

EATL is a rare and aggressive peripheral T-cell lymphoma, accounting for less than 1% of all NHL cases (8). This neoplasm originates from the malignant transformation of intraepithelial lymphocytes and comprises two subtypes. Classic EATL (formerly type I) is strongly associated with celiac disease and occurs predominantly in Western populations. Morphologically, classic EATL is characterized by pleomorphic tumor cells-typically a mixture of medium-sized to large lymphocytes-frequently accompanied by a background mixed inflammatory infiltrate. The typical immunophenotype of classic EATL is CD3(+), CD30(+), with variable CD8 expression, and is usually negative for CD56. In contrast, monomorphic epitheliotropic intestinal T-cell lymphoma (MEITL, formerly type II EATL) shows no clear association with celiac disease, is more common in Asian populations, and consists of monomorphic small- to medium-sized cells usually without a prominent inflammatory background. Its characteristic immunophenotype includes expression of CD8 and CD56, while CD30 is typically negative (9–11). Both subtypes express T-cell markers (e.g., CD3) and cytotoxic markers (e.g., TIA-1). Therefore, positivity for CD30 and negativity for CD56 are key immunophenotypic features that help distinguish classic EATL from MEITL. Common complications of EATL include abdominal pain, nausea, vomiting, diarrhea, and malabsorption; with disease progression, it can lead to intestinal obstruction, hemorrhage, and perforation (11). EATL can also disseminate beyond the GI tract, most commonly involving abdominal lymph nodes, followed by involvement of the lung and mediastinal lymph nodes, liver (2–8%), and skin (12). Central nervous system involvement is exceedingly rare, and osseous involvement has been scarcely reported (13, 14). An international multicenter study of 62 patients with EATL found that only 3% had bone marrow involvement at diagnosis, with no specifically reported cases of skeletal (bone parenchymal) involvement (15).

The patient presented with abdominal pain in the absence of significant diarrhea and subsequently developed both gastric and small intestinal perforations. Notably, a spontaneous pathological fracture during hospitalization prompted an ultrasound-guided bone biopsy, which confirmed osseous involvement—an exceptionally rare manifestation. The diagnosis of EATL is challenging as there is no single gold-standard test, and its histopathological appearance is highly variable, often featuring a mixed inflammatory infiltrate (e.g., histiocytes, eosinophils, plasma cells) that may obscure the neoplastic cells. Diagnostic challenges arise from atypical clinical presentations, limitations in obtaining adequate biopsy samples, complex pathological features, and the presence of necrosis and an inflammatory background that can mask lymphoma cells (11, 16). Consequently, this case necessitated multiple biopsies before a definitive diagnosis could be established. This case underscores that in patients with persistent, unexplained gastrointestinal symptoms and recurrent perforations—particularly elderly individuals with known risk factors—repeated biopsies and a high index of clinical suspicion are crucial. Furthermore, early multidisciplinary collaboration is essential to facilitate timely diagnosis.

### The uniqueness of this case and its clinical implications

1. Recurrent perforation and diagnostic challenges: The patient experienced two gastrointestinal perforations (gastric and jejunal) at distinct sites within a two-month period, necessitating multiple surgical interventions. This strongly suggests that EATL has highly aggressive and multifocal growth characteristics. Tumor cells can infiltrate through the wall, easily leading to weakened intestinal walls and necrosis, which can trigger perforation. Although an early endoscopic biopsy demonstrated dense lymphocytic infiltration, a definitive diagnosis was not established at that time. This may be attributed to superficial sampling during endoscopic biopsy, uneven distribution of lesions (as seen in this case with scattered multiple nodules during surgery), or significant inflammatory necrosis background interfering with the identification of tumor cells. This case highlights that in elderly patients with unexplained, recurrent gastrointestinal perforations, a high suspicion for lymphoma (particularly of T-cell origin) must be maintained even if initial endoscopic biopsies are non-diagnostic. In such scenarios, obtaining sufficient tissue for diagnosis may require deeper biopsies (e.g., endoscopic deep biopsy or ultrasound-guided core biopsy) or surgical exploration.
2. Hidden bone metastasis and differential diagnosis: The patient experienced right lower limb and knee pain early in the disease course (6 months prior to hospitalization), which was initially attributed to her long-standing rheumatoid arthritis. Patients with rheumatoid arthritis (RA) have a well-documented, significantly elevated risk of developing lymphoma compared to the general population This increased risk is primarily attributed to the chronic, systemic inflammatory state inherent to RA itself (17). Furthermore, while tofacitinib treatment has been associated with an elevated risk of lymphoma (18), there is currently no evidence suggesting that leflunomide induces lymphoma. Even after hospitalization, the MRI of the hip and knee joints revealed femoral lesions, but the initial impression leaned towards infection or an indeterminate tumor, without immediate connection to lymphoma. It was not until a spontaneous femoral fracture occurred that a targeted biopsy confirmed the diagnosis of lymphoma bone metastasis. This highlights the insidious nature of EATL bone metastasis. Its symptoms can be masked by or attributed to coexisting conditions such as RA, and early imaging findings are often non-specific. Therefore, in patients diagnosed with EATL, careful assessment of the skeletal system during staging is warranted. In the presence of new or unexplained bone pain, pathological fractures, or elevated alkaline phosphatase-even if initial bone scans or local imaging are unrevealing-more sensitive modalities such as MRI or PET-CT should be considered. A biopsy of suspicious lesions should be performed when indicated to establish a definitive diagnosis.
3. Considerations for individualized treatment decisions: At the time of diagnosis, the disease was already stage IV (with osseous metastasis) in an elderly patient (69 years old) with poor performance status and concomitant respiratory and circulatory failure. Although surgery successfully addressed the acute complication of intestinal perforation, radical resection was neither indicated nor feasible given the disseminated nature of the disease. According to the NCCN guidelines, systemic treatment is the primary approach for stage IV lymphoma, with surgery only used to control local complications (8). However, the patient’s general condition at that time could not tolerate intensive chemotherapy. Therefore, after discussion by the MDT, a strategy focused on palliative and supportive treatment was adopted, including brace fixation for femoral fractures. This decision-making process underscores the complexity and need for individualized management in EATL, particularly for patients with advanced disease and poor performance status. The therapeutic goals must balance disease control, symptom palliation, and quality of life.

EATL is a rare lymphoma that is highly aggressive and has a poor prognosis. This case emphasizes the following key points through the presentation of a complex clinical course characterized by recurrent gastrointestinal perforation and delayed bone metastasis:

1. Raise awareness: For unexplained, recurrent acute abdomen (especially perforation), the possibility of EATL should be considered, even if the patient has no typical history of celiac disease.
2. In-depth diagnosis: When diagnosis is difficult, active multidisciplinary collaboration should be sought, and clear pathology should be obtained through repeated, deep, or surgical biopsies. Comprehensive staging examinations are crucial for suspected cases.
3. Attention to metastasis: Recognizing that EATL can metastasize to rare sites such as bone, clinicians need to remain vigilant for related symptoms.
4. Individualized treatment: Treatment strategies should be based on precise staging, the patient’s physical condition, and complications, jointly formulated by the MDT, possibly involving a comprehensive application of surgery (to manage complications), chemotherapy (e.g., anthracycline-containing regimens), targeted therapy, or supportive care.

The aggressive nature and frequent chemorefractoriness of T-cell lymphomas, combined with the poor nutritional status often present at diagnosis, contribute to a poor prognosis and frequently limit intensive treatment options. Diagnostic and therapeutic delays further contribute to high mortality, as evidenced by cohort studies reporting a median survival of approximately 7 months in patients with EATL (19–21). Owing to the rarity of EATL and a paucity of prospective studies, no standardized treatment regimen exists. Current management strategies primarily involve chemotherapy, with surgery typically reserved for managing complications. When available, enrollment in clinical trials is the preferred option. In the absence of suitable trials, chemotherapy regimens such as CHOP, CHOEP, dose-adjusted EPOCH, or hyper-CVAD may be considered (8). Recent studies have demonstrated notable efficacy for brentuximab vedotin in CD30-positive cases, underscoring the importance of CD30 testing to inform therapeutic decisions (22).

## Summary

Although the key clinicopathological features of EATL have been delineated, the rarity of the disease has impeded research progress, resulting in a paucity of evidence-based guidelines to inform treatment decisions. Consequently, greater attention from the scientific community is warranted to establish standardized diagnostic and therapeutic protocols. Early diagnosis and standardized management are pivotal for improving the prognosis of patients with EATL.

## Data Availability

The original contributions presented in the study are included in the article/supplementary material, further inquiries can be directed to the corresponding authors.
